# P-273. User and Community Reflections and Recommendations on the Promise of HIV Molecular Epidemiology

**DOI:** 10.1093/ofid/ofaf695.494

**Published:** 2026-01-11

**Authors:** Juan D Patino-Mateus, Olakunle Ogunbayo, Aimee Graciela Rivera-Solis, Adaiah Soibi-Harry, Claudia E Ordóñez, Julian S Ramon, Masonia Traylor, Amalia Aldredge, David Folkes, Jenna Gettings, Daniel Mauck, Latasha Terry, Eric Rangel, Anandi N Sheth, Jessica Sales, Jane Y Scott, Carlos S Saldana

**Affiliations:** Emory University School of Medicine, Atlanta, GA; Emory Rollins School of Public Health, Atlanta, Georgia; Emory University School of Medicine, Atlanta, GA; Emory University, Atlanta, Georgia; Emory University, Atlanta, Georgia; Emory University School of Medicine, Atlanta, GA; Lady Burgandy, Atlanta, Georgia; Emory University, Atlanta, Georgia; ThriveSS, Atlanta, Georgia; Centers for Disease Control and Prevention, Atlanta, Georgia; Georgia Department of Public Health, Atlanta, Georgia; Georgia Department of Public Healtb, Atlanta, Georgia; Latino Linq, Atlanta, Georgia; Emory University School of Medicine, Atlanta, GA; Emory University, Rollins School of Public Health, Atlanta, Georgia; Emory University School of Medicine, Fulton County Board of Health, Atlanta, Georgia; Emory University School of Medicine, Atlanta, GA

## Abstract

**Background:**

HIV Molecular Epidemiology (HME) uses viral genetic pol sequences to identify transmission networks and guide public health responses. While HME offers promises for addressing rapid or ongoing HIV transmission, concerns about privacy, informed consent, and potential criminalization have limited community trust and hindered widespread implementation. This study aims to explore the perceptions of public health officials (PHOs) and priority populations to inform community-centered HME practices.

**Figure:**
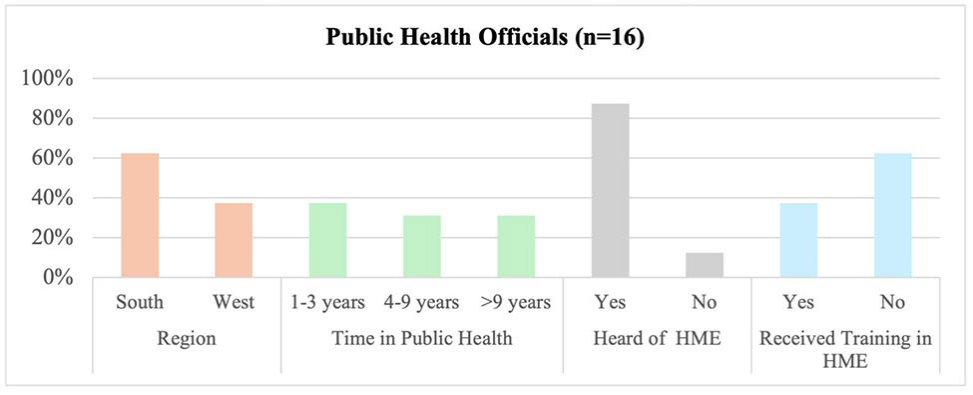
Characteristics of public health officials (PHOs) HME = HIV molecular epidemiology

**Figure:**
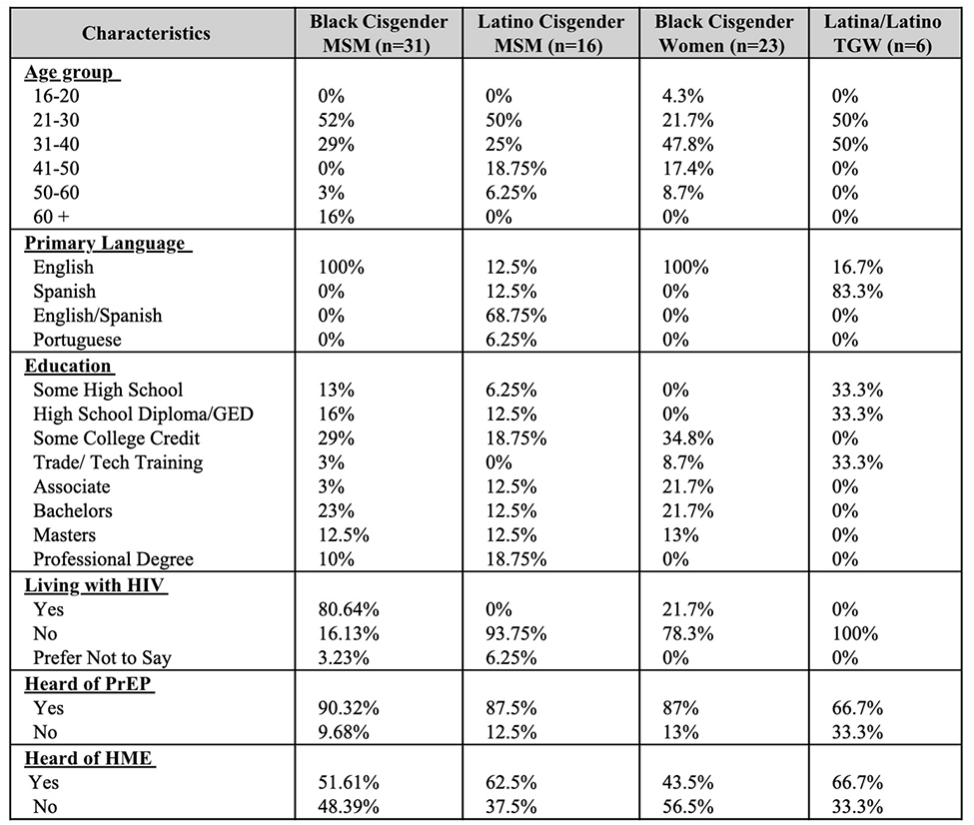
Demographics of priority populations MSM = men who have sex with men, TGW = transgender women, PrEP = pre-exposure prophylaxis, HME = HIV molecular epidemiology

**Methods:**

We conducted virtual focus groups discussions (FGD) with PHOs and Black cisgender women, Black and Latino men who have sex with men (MSM), and Latina/Latino transgender women (TGW) in Metropolitan Atlanta using a community-engaged, multi-method design. Participants were recruited in partnership with local trusted community-based organizations and prior to each FGD, watched a video explaining HME. Discussions explored (1) perceptions on HME, (2) key attributes for PHOs involved in HME, (3) HME-related training for PHOs, (4) HME communication practices, and (5) ethical HME practice. Data were analyzed using descriptive statistics and reflexive thematic analysis.

**Figure:**
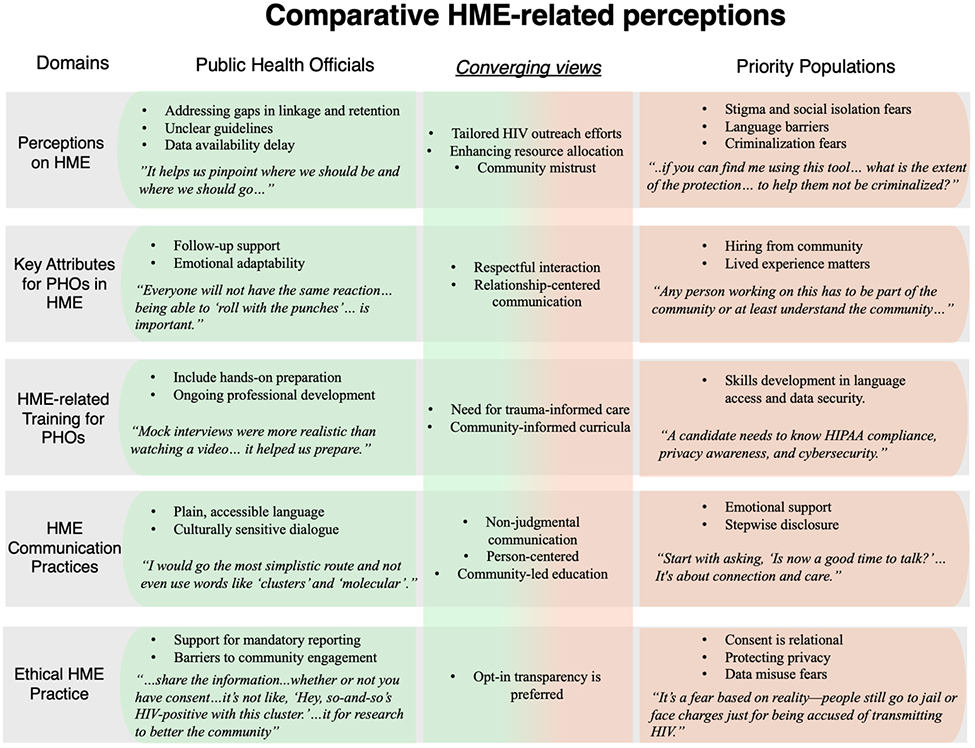
Key considerations that emerged in discussions about HME with public health officials (PHOs) and priority populations The first column represents the five domains defined for the thematic analysis of qualitative data. The following columns list key HME-related perceptions and perspectives of PHOs and members of priority populations who participated in the research, organized by qualitative domains, with representative quotes in italics and converging views placed in between

**Results:**

We conducted 18 FGDs among 92 research participants (Figure 1, Table 1). Our data showed that (1) HME holds promise for outreach and linkage but must overcome community mistrust tied to privacy, stigma, and criminalization fears; (2) lived experience, cultural understanding, and empathy must guide HME; (3) PHOs’ HME training must integrate trauma-informed care, privacy protections, community voices, and continuous skill-building; (4) simple, relatable communication and relationship-building are necessary for meaningful community engagement with HME, and that (5) ethical HME requires consent and dialogue, given existing fears about data misuse (Figure 2).

**Conclusion:**

Community-centered HME depends on transparency, individual agency, cultural sensitivity, engagement, and commitment. Strengthening trust requires centering lived experience, protecting privacy, enhancing current PHO training, and communicating HME-related information clearly. These elements are necessary for the successful implementation of HME in outreach, linkage, and public health equity.

**Disclosures:**

All Authors: No reported disclosures

